# Preclinical immunological characterization of rademikibart (CBP-201), a next-generation human monoclonal antibody targeting IL-4Rα, for the treatment of Th2 inflammatory diseases

**DOI:** 10.1038/s41598-023-39311-2

**Published:** 2023-07-31

**Authors:** Limin Zhang, Ying Ding, Qingjian Wang, Wubin Pan, Zheng Wei, Paul A. Smith, Xin Yang

**Affiliations:** 1 Suzhou Connect Biopharmaceuticals, Ltd, East R&D Building, 6 Beijing West Road, Taicang, 214500 China; 2 Connect Biopharma LLC, 12265 El Camino Real, San Diego, CA 92130 USA

**Keywords:** Cytokines, Asthma, Skin diseases, Antibody therapy

## Abstract

Rademikibart (CBP-201) is a next-generation human monoclonal antibody targeting IL-4Rα, undergoing evaluation in Phase 2 clinical trials for the treatment of moderate-to-severe Th2 inflammatory diseases. We report the immunological characterization of rademikibart. Rademikibart and dupilumab were associated with K_D_ of 20.7 pM and 45.8 pM, respectively, when binding to distinct human IL-4Rα epitopes. Rademikibart did not bind to IL-4Rα from other species. Rademikibart inhibited IL-4 and IL-13-mediated STAT6 signaling (mean ± SD IC_50_: 7.0 ± 2.5 and 6.6 ± 1.5 ng/mL, respectively), TF-1 cell proliferation (IC_50_: 8.0 ± 1.6 and 9.7 ± 0.8 ng/mL, respectively) and TARC production in PBMCs (IC_50_: 59.2 ± 3.9 and 13.5 ± 0.2 ng/mL, respectively). Rademikibart versus dupilumab was more potent in the STAT6 assays (IL-4, p < 0.01; IL-13, p = 0.03), with non-significant trends towards greater potency in the TF-1 cell assays (IL-4, p = 0.09; IL-13, p = 0.20), and similar potency in the TARC assays. In experiments with mice expressing human IL-4Rα and IL-4, rademikibart and dupilumab demonstrated similar potency; both monoclonal antibodies eliminated IL-4 (p < 0.0001) and IL-13 (p < 0.05) mediated B cell activation in vitro and ovalbumin-induced IgE (p < 0.01) and eosinophilic lung infiltration (p < 0.0001) in vivo. In Th2-stimulated human skin explants, rademikibart rapidly downregulated IL-4, IL-13, and TARC gene expression, with greater effectiveness than dupilumab for IL-4 (p < 0.01) and a non-significant trend towards superiority for IL-13. In summary, rademikibart bound to a distinct IL-4Rα epitope with high affinity and demonstrated reductions in Th2 inflammatory biomarkers with at least similar and potentially superior potency to dupilumab*.*

## Introduction

T helper-2 (Th2) inflammatory diseases including asthma and atopic dermatitis (AD) affect a large proportion of the worldwide population, with prevalence varying by age, clinical definitions, country, and sampling methods^1–14^. Among adults and children in the US, prevalence estimates of 8.4% and 5.8% have been reported for asthma, and 7.2% and 12.5% for AD, respectively^9–14^. In adults, the estimated global prevalence of asthma symptoms (8.6%) is similar per continent (Latin America 7.6%, Europe 10.7%) with some variation by country (Australia 27.4%)^1^, while AD may be most prevalent in Sweden (11.5%)^8^.

Asthma and AD are often inadequately controlled^15–17^. In the US, millions of work/school days are lost each year due to asthma attacks, which can lead to hospital admission and even death^12,15,18^. AD is characterized by recurrent eczematous lesions and intense pruritus, resulting in disturbed sleep, embarrassment, and increased risk of anxiety, depression, and suicidal ideation^10,11,19–22^. Use of medications for AD may be limited by adverse events and inefficacy^17,23^.

Two proinflammatory Th2 cytokines, interleukin (IL)-4 and IL-13, and their cognate receptor (IL-4Rα), are key drivers of AD and asthma pathogenesis^24–26^. IL-4 and IL-13 are overexpressed in both diseases^24,25,27–29^. Other biomarkers of Th2 inflammatory diseases include thymus and activation-regulated chemokine (TARC), which recruits Th2 cells to inflammatory sites^24,30,31^, and over-secretion of IgE by B cells^24,25^. IL-4 and IL-13 promote IgE synthesis^24,25^. Monoclonal antibody therapies that selectively target IL-4Rα or IL-13 have demonstrated favorable efficacy, pharmacodynamics, safety, and tolerability in clinical trials and real-world studies of Th2-driven inflammatory diseases, including AD and asthma^24,32–40^.

Rademikibart (CBP-201), a next-generation monoclonal antibody targeting IL-4Rα, is undergoing evaluation in Phase 2 clinical trials in patients with moderate-to-severe Th2 inflammatory diseases, including AD (NCT04444752; NCT05017480) and persistent asthma (NCT04773678). Here, we report the first immunological characterization of rademikibart, assessed in vitro in a diverse collection of cellular systems, in vivo in a mouse model of Th2 inflammatory disease, and ex vivo in human skin.

## Results

### Rademikibart binds with high affinity to human IL-4Rα via a distinct epitope

**Figure 1:**
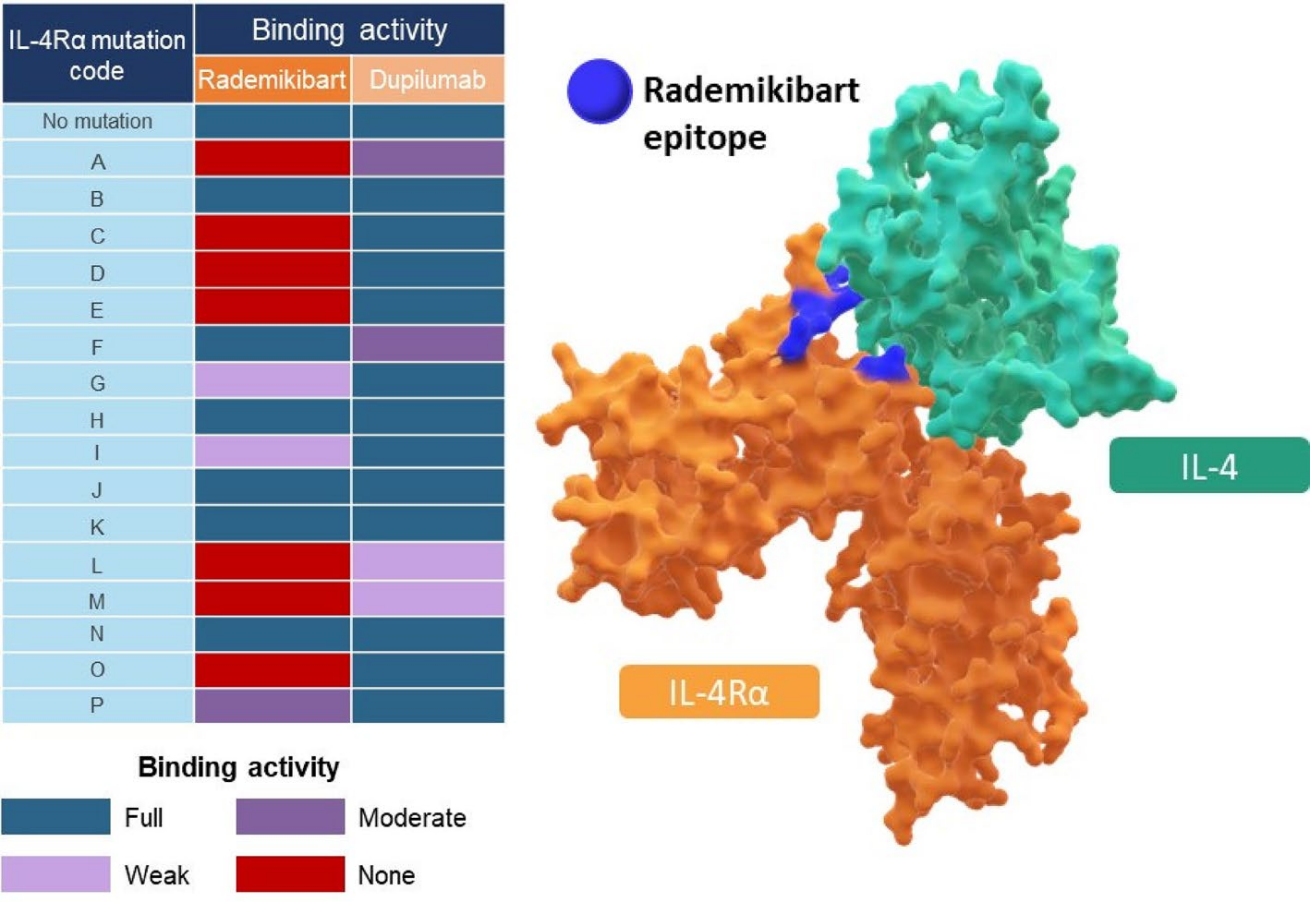
Rademikibart and dupilumab bind to distinct epitopes on human IL-4Rα. As shown in the table, mutation of amino acid residues (coded as A, C, D, E, L, M, and O), which are crucial for IL-4 binding, had little effect on the affinity for dupilumab but abolished binding of rademikibart, when assessed by ELISA. A more detailed version of the table (detailing the amino acid mutations associated with each mutation code letter and EC_50_ values) is shown in Supplementary Fig. 1. Binding epitopes for rademikibart on human IL-4Rα are shown in the figure, based on the mutational analysis*. *EC*_*50*_ half maximal effective concentration. *Mutation G is based on mutation of two amino acids; a stable single mutant could not be created, therefore both residues are highlighted blue in the figure.

Rademikibart and dupilumab were associated with K_D_ of 20.7 pM and 45.8 pM, respectively, for binding to human IL-4Rα (Table 1). As shown by mutational analysis, rademikibart and dupilumab bound to distinct epitopes on human IL-4Rα (Fig. 1 and Supplementary Fig. 1). Mutation of amino acid residues that are crucial for IL-4 binding had little effect on the affinity for dupilumab but abolished binding of rademikibart.

**Table 1:** Rademikibart binds with high affinity to human IL-4Rα.

Protocol	Immobilized protein	Analyte	1:1 binding		
			k_a_ (1/Ms)	k_d_ (1/s)	K_D_ (pM)
A	sIL-4Rα	Rademikibart	1.54 × 10^7^	3.19 × 10^−4^	20.7
	sIL-4Rα	Dupilumab	7.71 × 10^6^	3.53 × 10^−4^	45.8
B	Rademikibart	sIL-4Rα	6.51 × 10^5^	2.92 × 10^−4^	448
	Dupilumab	sIL-4Rα	6.41 × 10^5^	3.31 × 10^−4^	516

Two surface plasmon resonance protocols were used. *Protocol A:* Histidine tagged sIL-4Rα was immobilized onto a treated CM5 sensor chip, to capture rademikibart or dupilumab analytes at different concentrations. *Protocol B:* Rademikibart or dupilumab were immobilized onto IgG Fc CM5 sensor chips, to capture sIL-4Rα analyte at different concentrations.

*k*_*a*_ association rate constant, *k*_*d*_ dissociation rate constant, *K*_*D*_ equilibrium dissociation constant, *sIL-4Rα* soluble IL-4Rα.

### Rademikibart is specific for human IL-4Rα

**Figure 2:**
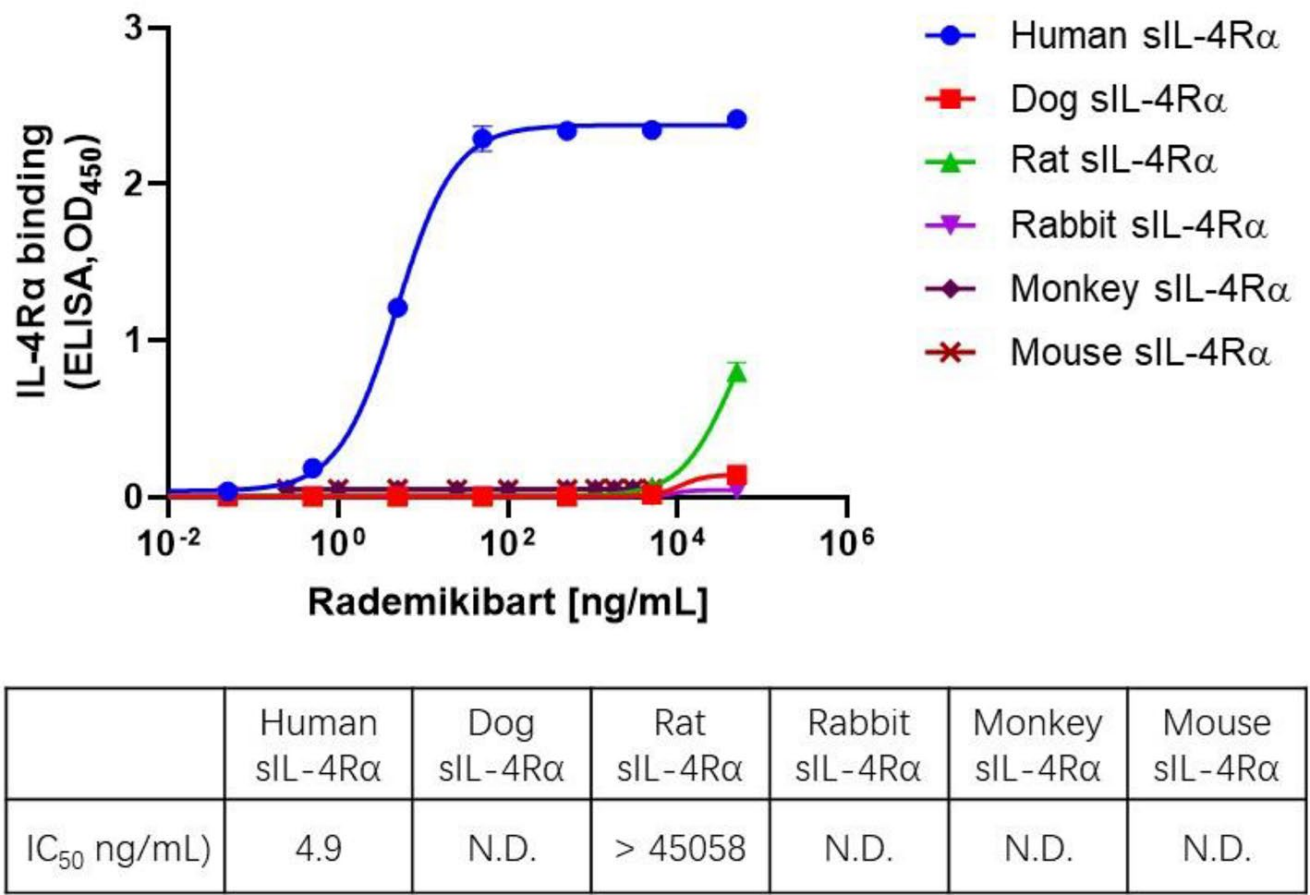
Rademikibart is specific for human IL-4Rα. Rademikibart bound to human sIL-4Rα with a K_D_ of 106 pM and IC_50_ of 4.9 ng/mL, but did not cross react with sIL-4Rα of other species (two or three replicates per species). *IC*_*50*_ half maximal inhibitory concentration, *K*_*D*_ equilibrium dissociation constant, *N.D.* not detected, *sIL-4Rα* soluble IL-4Rα.

Cross-species ELISA revealed that rademikibart bound to human sIL-4Rα with a K_D_ of 106 pM and IC_50_ of 4.9 ng/mL, but did not cross react with IL-4Rα isolated from monkey, mouse, dog, rat, or rabbit (Fig. 2).

### Rademikibart inhibits cytokine-induced intracellular signaling

In the human embryonic kidney (HEK) Blue™ reporter cell assay, incubation with rademikibart resulted in concentration-dependent inhibition of STAT6 intracellular signaling, which had been activated by IL-4 or IL-13 (Table 2 and Supplementary Fig. 2). When compared head-to-head, rademikibart was associated with significantly greater STAT6 inhibition than with dupilumab in cells stimulated with IL-4 (mean ± standard deviation [SD] IC_50_: 7.0 ± 2.5 vs 9.9 ± 2.7 ng/mL; p < 0.01) and IL-13 (6.6 ± 1.5 vs 9.7 ± 2.5 ng/mL; p = 0.03).

**Table 2:** Rademikibart inhibits cytokine-induced intracellular STAT6 signaling, TF-1 cell proliferation, and TARC secretion, generally with trends towards greater potency than dupilumab. Curve diagrams are shown in Supplementary Fig. 2.

Cytokine stimulation	Monoclonal antibody	IC_50_ (ng/mL)					
		STAT6 signaling	p value	TF-1 cell proliferation	p value	TARC secretion	p value
IL-4	Rademikibart	7.0 ± 2.5	< 0.01	8.0 ± 1.6	0.09	59.2 ± 3.9	0.11
	Dupilumab	9.9 ± 2.7		10.8 ± 1.1		65.2 ± 5.5	
IL-13	Rademikibart	6.6 ± 1.5	0.03	9.7 ± 0.8	0.20	13.5 ± 0.2	0.41
	Dupilumab	9.7 ± 2.5		12.0 ± 2.4		10.8 ± 2.7	

IC_50_ expressed as mean ± standard deviation. *p* values for rademikibart versus dupilumab. Intracellular signaling was assessed using the HEK Blue™ STAT6 reporter cell assay**.** TF-1 cell proliferation was quantified using a cell counting kit-8**.** TARC secretion from PBMCs was quantified by ELISA.

*HEK* human embryonic kidney, *IC*_*50*_ half maximal inhibitory concentration, *PBMC* primary human peripheral blood mononuclear cells.

### Rademikibart binds strongly to TF-1 cells, inhibiting cytokine-induced TF-1 cell proliferation

Rademikibart bound strongly to TF-1 cells (Supplementary Fig. 3). Moreover, rademikibart inhibited cytokine-induced TF-1 cell proliferation in a concentration-dependent manner, with non-significant trends towards greater potency than dupilumab following stimulation with IL-4 (mean ± SD IC_50_: 8.0 ± 1.6 vs 10.8 ± 1.1 ng/mL; p = 0.09) and IL-13 (9.7 ± 0.8 vs 12.0 ± 2.4 ng/mL; p = 0.20) (Table 2 and Supplementary Fig. 2).

### Rademikibart inhibits cytokine-induced TARC release

Primary human peripheral blood mononuclear cells (PBMCs), when activated in vitro with either IL-4 or IL-13, released TARC into the culture supernatant. Rademikibart and dupilumab demonstrated similar potency when inhibiting TARC release stimulated by IL-4 (mean ± SD IC_50_: 59.1 ± 1.2 vs 65.2 ± 1.1 ng/mL; p = 0.11) and IL-13 (13.6 ± 1.1 vs 10.6 ± 1.0 ng/mL; p = 0.41) (Table 2 and Supplementary Fig. 2).

### Rademikibart inhibits cytokine-induced B cell activation

**Figure 3:**
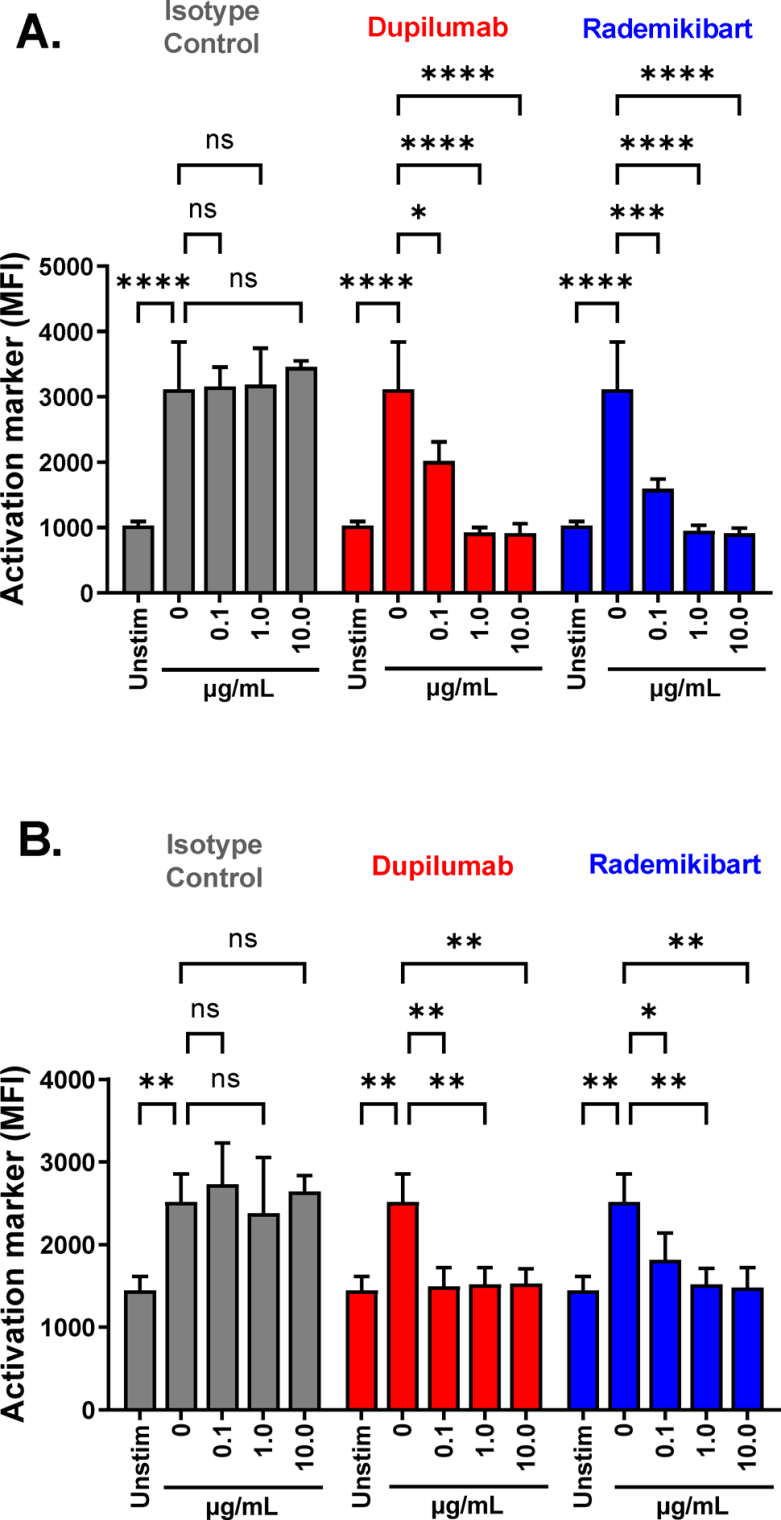
Rademikibart inhibits cytokine-induced B cell activation. Splenocytes were isolated from double humanized IL-4/IL-4RA (B-hIL4/hIL4RA) mice. Incubation with rademikibart resulted in concentration-dependent inhibition of (**A**) IL-4-induced CD23 and (**B**) IL-13-induced MHCII expression, analyzed by flow cytometry. ‘Unstim’ shows B cell activation marker expression without cytokine stimulation. The ‘0 μg/mL’ monoclonal antibody groups served as positive controls. No statistically significant differences in B cell activation marker expression were observed when comparing each concentration of rademikibart vs dupilumab. N = 3 mice per group. Data are expressed as mean ± standard deviation. One-way ANOVA and post hoc test (*p < 0.05, **p < 0.01, ***p < 0.001, ****p < 0.0001, *ns* not significant).

IL-4 and IL-13 stimulation of splenocytes, isolated from commercially available genetically modified mice (double humanized IL-4/IL-4RA [B-hIL4/hIL4RA]), resulted in 3.0-fold and 1.7-fold increases in CD23 and MHCII expression, respectively. Large, statistically significant decreases in the expression of both molecules were observed with all concentrations of rademikibart (0.1, 1.0, and 10.0 μg/mL; 3 mice per group); CD23 (p < 0.0001) and MHCII (p < 0.05) returned to unstimulated levels with rademikibart 1.0 μg/mL and 0.1 μg/mL (Fig. 3). B cell responses to rademikibart were similar in magnitude to those with dupilumab.

### Rademikibart inhibits ovalbumin-induced Th2 allergy in a mouse model

**Figure 4:**
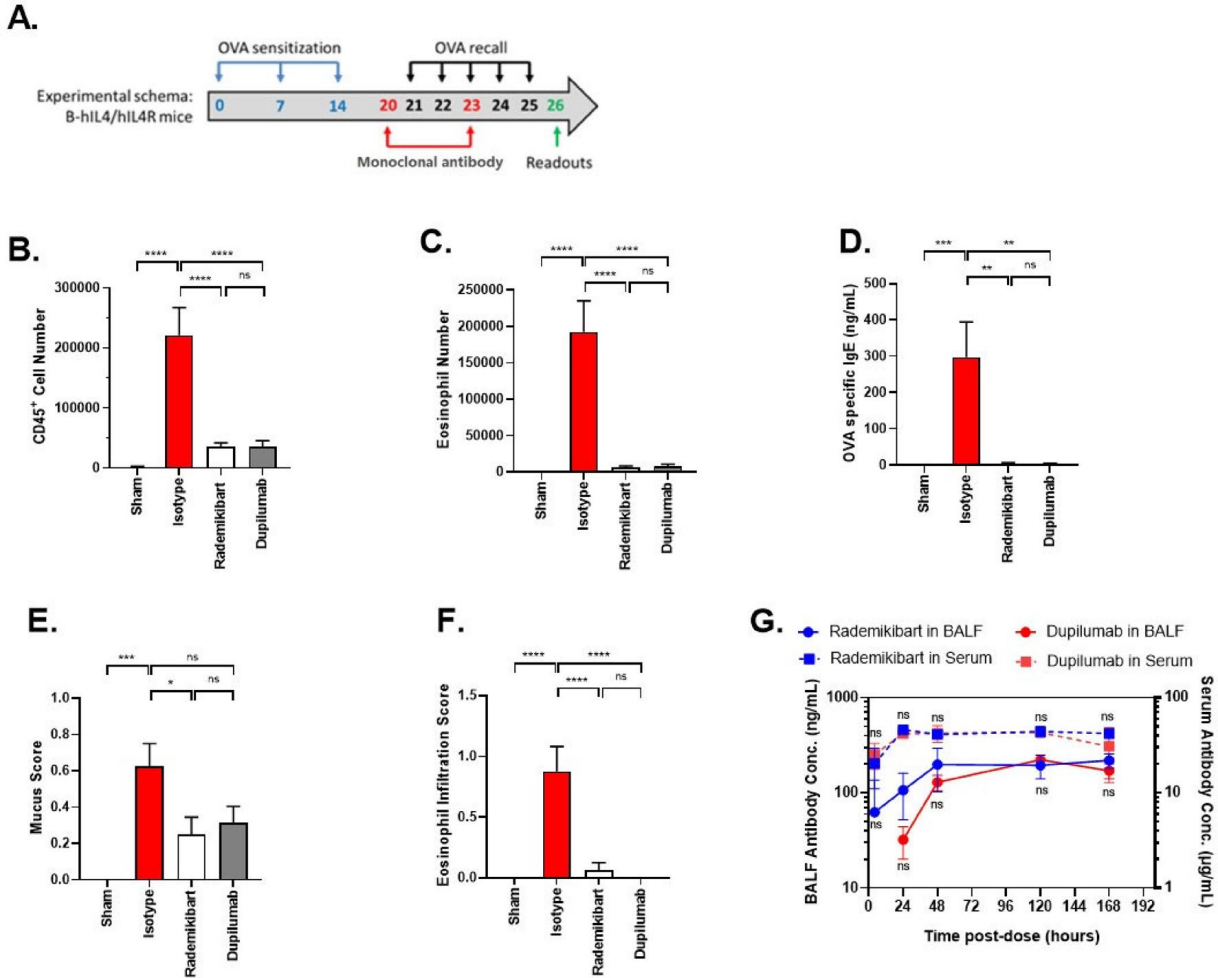
Rademikibart ameliorates Th2-driven allergic responses. (**A**) Schematic design of the ovalbumin-induced lung inflammation mouse model (time is shown in days), with humanized IL-4/IL-4RA (B-hIL4/hIL4RA) mice (N = 8 per group). In the mouse model, subcutaneous administration of rademikibart or dupilumab (25 mg/kg; on Days 20 and 23) resulted in reductions (on Day 26) in the total number of (**B**) CD45+ hematopoietic cells and (**C**) eosinophils in alveolar lavage fluid, (**D**) ovalbumin-specific serum IgE concentration, (**E**) airway mucus score, and (**F**) eosinophil infiltration. (**G**) In wildtype mice (N = 3 per time point, per treatment), within 48 h post-treatment (5 mg/kg), there was a non-significant trend towards higher rademikibart-specific antibody concentrations than for dupilumab in bronchoalveolar lavage fluid (BALF), despite similar concentrations in serum (BALF antibody concentration for dupilumab at 4 h post-treatment was lower than the test limit). No statistically significant differences were observed with rademikibart vs dupilumab in the mouse model or in the wildtype mice. Data are expressed as mean ± standard error of the mean. One-way ANOVA and post hoc test (*p < 0.05, **p < 0.01, ***p < 0.001, ****p < 0.0001, *ns* not significant).

Subcutaneous administration of rademikibart or dupilumab (25 mg/kg) to B-hIL-4/hIL-4RA mice during the OVA recall phase ameliorated several Th2-driven allergic responses (8 mice per treatment; Fig. 4A–F). Compared with the hIgG4 isotype control, both treatments almost completely eliminated the increased number of immune cells (CD45+ hematopoietic cells and eosinophils; p < 0.0001) and ovalbumin-specific IgE concentration in the blood (p < 0.01), as well as eosinophil infiltration in lung tissue (p < 0.0001). Airway mucus scores decreased by 60% with rademikibart treatment, reaching statistical significance versus the hIgG4 isotype control (p < 0.05), whereas the 50% reduction with dupilumab was not significant either versus the control or rademikibart (Fig. 4E). Based on medication-specific antibody concentrations in wildtype mice (3 mice per time point, per treatment [5 mg/kg]), a non-significant trend towards greater quantity of rademikibart than dupilumab was detected in bronchoalveolar lavage fluid (BALF) within 48 h post-treatment, despite similar blood exposure (Fig. 4G).

### Rademikibart downregulates expression of inflammatory genes in Th2-stimulated human skin

**Figure 5:**
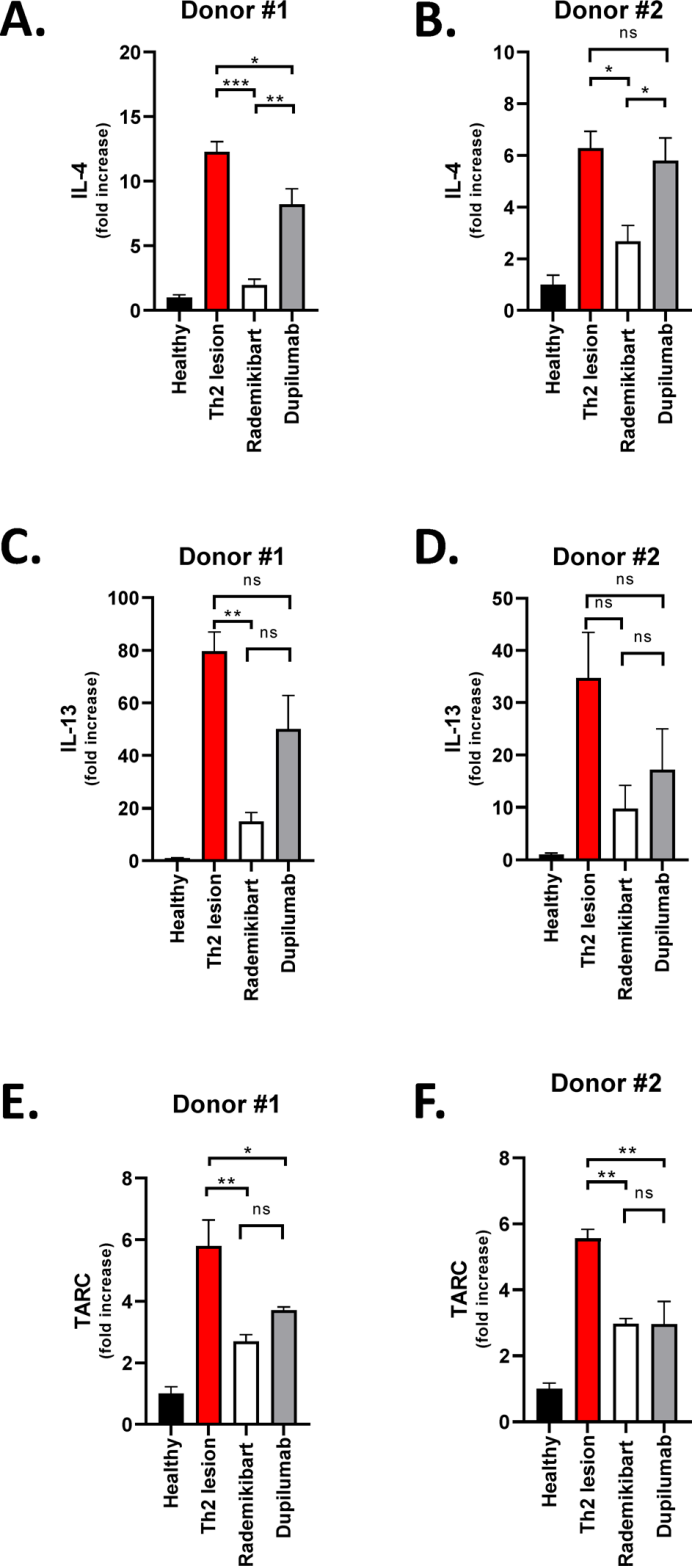
Rademikibart downregulates expression of inflammatory genes in Th2-stimulated human skin explants from two independent donors. Inflammatory genes investigated in Donors #1 and #2, respectively, were (**A**,**B**) IL-4, (**C**,**D**) IL-13, and (**E**,**F**) TARC. ‘Healthy’ shows gene expression without ex vivo Th2 stimulation or exposure to either monoclonal antibody. ‘Th2 lesion’ shows elevated gene expression in healthy skin explants exposed to a Th2 stimulation cocktail alone. ‘Rademikibart’ and ‘dupilumab’ show the effects of ex vivo pretreatment with each monoclonal antibody (10 μg/mL) on Th2-stimulated skin. Data are expressed as mean ± standard error of the mean. Three or four replicates per donor. One-way ANOVA and post hoc test (*p < 0.05, **p < 0.01, ***p < 0.001, ****p < 0.0001, *ns* not significant).

Following Th2 stimulation, IL-4, IL-13, and TARC gene expression was upregulated in healthy human skin from two independent donors (Fig. 5). Rademikibart (10 μg/mL) downregulated the expression of all three genes in the Th2-stimulated explants. When compared head-to-head with dupilumab (10 μg/mL), the reduction in IL-4 expression was significantly greater with rademikibart, and there was a non-significant trend towards greater reduction in IL-13 with rademikibart (Fig. 5). For instance, in explants from Donor #1 (three or four explants analyzed per group), IL-4 gene expression was 12.3-fold higher following Th2 stimulation versus unstimulated tissue (p < 0.001), and decreased to 2.0-fold versus 8.2-fold higher with rademikibart versus dupilumab pretreatment, respectively (p < 0.01 comparing the two monoclonal antibodies), than in unstimulated tissue.

## Discussion

This is the first description of the immunological characterization of rademikibart, a next-generation monoclonal antibody targeting human IL-4Rα. We demonstrated that rademikibart bound with high affinity to human IL-4Rα, without cross-reaction to other mammalian IL-4Rα, resulting in potent downregulation of Th2-driven inflammation in vitro, in vivo (transgenic mice), and ex vivo (human skin). All experiments (except for cross-species IL-4Rα binding of rademikibart) were head-to-head with dupilumab, a currently marketed IL-4Rα targeting therapy for Th2-driven inflammatory diseases (AD, asthma, chronic rhinosinusitis with nasal polyposis, eosinophilic esophagitis, and prurigo nodularis)^32,35–37,40,41^. Notably, these head-to-head experiments indicated improved target engagement properties—based on the binding affinity of rademikibart (K_D_ 20.7 pM) for human IL-4Rα, via an epitope distinct from dupilumab (K_D_ 45.8 pM), for which some epitope mapping has previously been published^42,43^—and resulted in potent inhibition of Th2-driven inflammation that was at least comparable and potentially superior to dupilumab.

The improvements in Th2-driven inflammatory responses by rademikibart were demonstrated using a diverse collection of in vitro cellular systems. IL-4 and IL-13, when engaged with their cognate receptor, trigger intracellular activation of the Janus kinase (JAK)-signal transduction and activator of the STAT6 pathway^44^, and are growth factors for the TF-1 cell line^45^. Another molecule, TARC, recruits Th2 cells to inflammatory sites and is a clinical biomarker of AD activity^30,31^. All rademikibart-induced in vitro reductions in Th2-driven inflammation (IL-4 and IL-13-mediated JAK-STAT intracellular signaling, TF-1 cell proliferation, and TARC production) were concentration-dependent. Compared with dupilumab, rademikibart was more potent at inhibiting STAT6 signaling, with non-significant trends towards greater potency at reducing TF-1 cell proliferation, and similar potency when downregulating TARC production.

Given the highly selective binding of rademikibart to human IL-4Rα, and not to IL-4Rα from any other mammalian species, experiments were conducted with genetically modified mice; expression of mouse IL-4Rα and its IL-4 ligand was fully replaced by the human proteins. Beyond in vitro T cell-mediated Th2 responses, rademikibart resulted in concentration-dependent inhibition of IL-4 and IL-13-mediated activation of primary B cells, with a potency similar to dupilumab, in experiments with murine splenocytes. The in vivo ovalbumin-induced Th2 allergy mouse model is well characterized and validated^46,47^. During the allergen (ovalbumin) recall inflammatory response, subcutaneous administration of rademikibart significantly ameliorated accumulation of CD45+ hematopoietic cells and eosinophils in the blood. Allergen-specific IgE blood titers were also significantly reduced, consistent with the in vitro B cell inhibition data. Similarly, blinded histological analysis of lung target tissue revealed significant reductions in the amount of airway mucus and eosinophil infiltration. Interestingly, despite similar blood exposure, there was a trend for rademikibart to distribute more rapidly into BALF than dupilumab in wildtype mice. Whether this tendency towards enhanced lung tissue exposure to rademikibart corresponded to the small difference in mucus score with rademikibart (60% decrease) versus dupilumab (50% decrease) in the mouse model remains unclear (the 60% reduction in mucus score with rademikibart reached statistical significance versus hIgG4 isotype control (p < 0.05), whereas the 50% reduction with dupilumab was not significant versus both hIgG4 isotype control and rademikibart).

Analyses of ex vivo Th2-stimulated human skin corroborated the in vitro and in vivo findings. Rademikibart rapidly downregulated IL-4, IL-13 and TARC expression in skin explants from two independent donors. Intriguingly, in these head-to-head ex vivo experiments, rademikibart was significantly more effective than dupilumab at inhibiting IL-4 gene expression, with a non-significant trend towards superiority for IL-13 gene expression, when both treatments were administered at the same concentration (10 μg/mL). This finding may be related to greater soft tissue exposure with rademikibart than dupilumab, observed in the mouse model.

In summary, this manuscript represents the first immunological characterization of rademikibart, a highly potent and selective monoclonal antibody targeting human IL-4Rα. Reductions in inflammatory responses were observed in vitro (in intracellular STAT6 signaling, TF-1 cell proliferation, TARC release, and B cell activation), in vivo (in eosinophil infiltration and IgE release), and ex vivo (in IL-4, IL-13, and TARC gene expression). In these experiments, rademikibart was at least comparable, and potentially superior in vitro and ex vivo, to the currently approved treatment targeting human IL-4Rα. Rademikibart is currently being evaluated in Phase 2 clinical trials for the treatment of moderate-to-severe AD (NCT04444752, NCT05017480) and persistent asthma (NCT04773678).

## Materials and methods

For the experiments reported here, the source of the investigational treatment (rademikibart) was the same as in the Phase 2 clinical trials. Rademikibart was discovered by screening potential candidate molecules for T cell modulatory activity^48^. Head-to-head experiments of rademikibart were in comparison with commercially available dupilumab (Dupixent^®^) or an in-house dupilumab analog produced by transient transfection of HEK293 cells (Thermo Fisher Scientific, USA). Two vectors were used to produce the analog, pFUSE-CHIg-hG4 and pFUSE2-CLIg-hK (InvivoGen, USA). Dupilumab heavy and light chain V-region sequences were inserted into pFUSE-CHIg-hG4 and pFUSE2-CLIg-hK, respectively. HEK293 cells were transfected with the two vectors at a ratio of 1:1.

### Binding affinity to IL-4Rα

Two surface plasmon resonance protocols were used. *Protocol A:* Histidine tagged sIL-4Rα was immobilized onto a treated CM5 sensor chip, to capture rademikibart or dupilumab analytes at different concentrations. *Protocol B:* Rademikibart or dupilumab were immobilized onto IgG Fc CM5 sensor chips, to capture sIL-4Rα analyte at different concentrations. Data were acquired with the Biacore 8K (GE, USA) and equilibrium dissociation constant (K_D_), association rate constant (k_a_), and dissociation rate constant (k_d_) calculated.

### IL-4Rα epitope mapping

Site-specific mutations and alanine scanning mutations were incorporated into sIL-4Rα to identify epitopes that bind to rademikibart or dupilumab antibodies. An in-house ELISA was used to evaluate the binding activity of rademikibart or dupilumab to the sIL-4Rα mutants using a Flex Station 3 (Molecular Devices, USA) and half maximal effective concentration (EC_50_) calculated. See the Supplementary Methods section for information about the in-house ELISA.

### Cross-species IL-4Rα binding

Binding of rademikibart to human, cynomolgus monkey, dog, rat, rabbit, and mouse IL-4Rα was evaluated using an in-house ELISA. Colorimetric change of TMB solution was detected using a Flex Station 3 and K_D_ calculated.

### Cytokine-induced intracellular signaling 

HEK Blue™ IL-4/IL-13 cells containing a STAT6 reporter gene (Invivogen, USA) were stimulated with 0.5 ng/mL IL-4 or 2.5 ng/mL IL-13, either alone or in the presence of rademikibart or dupilumab. As per the manufacturer’s instructions, STAT6 activation was quantified via secreted alkaline phosphatase hydrolysis of QUANTI-Blue™ substrate colorimetric change. Data were acquired with the Flex Station 3 and half maximal inhibitory concentration (IC_50_) calculated.

### Binding to TF-1 cells and cytokine-induced TF-1 cell proliferation

TF-1 human erythroid leukemia cells (ATCC, USA) were suspended in 45 μL FACS buffer (PBS with 0.5% BSA). For the TF-1 cell binding experiments, rademikibart or dupilumab were added to the solution (100 μg/mL) and, after incubating on ice for 30 min, the cells were washed twice and resuspended in 45 μL FACS buffer. Following the addition of goat anti-human IgG(H + L) Alexa Fluor 488 (Invitrogen, China), another 30-min incubation on ice and two more cell washes, rademikibart and dupilumab binding was detected by flow cytometry (BD Calibur).

For the TF-1 cell proliferation experiments, the cells were stimulated with IL-4 (0.5 ng/mL) or IL-13 (5 ng/mL; Sino Biological, China), followed by the addition of rademikibart or dupilumab. TF-1 cell proliferation was quantified after 72 h using a cell counting kit-8 (CCK-8, Beyotime, China) as per the manufacturer’s instructions. Data were acquired with the Flex Station 3 and IC_50_ calculated.

### Cytokine-induced TARC release

PBMCs (Sailybio, China) were stimulated with IL-4 (2 ng/mL) or IL-13 (1 ng/mL). Rademikibart or dupilumab were added to the culture for 72 h and supernatants collected. Human TARC concentrations were quantified by ELISA (Abcam, UK) as per the manufacturer’s instructions. Data were acquired with the Flex Station 3 and IC_50_ calculated.

### Cytokine-induced B cell activation 

B-hIL4/hIL4RA mice (Biocytogen, China) express human IL-4 and IL-4RA, replacing their murine counterparts^47^. Splenocytes were isolated from B-hIL4/hIL4RA mice and incubated with human IgG4 isotype control (CrownBio, USA), rademikibart or dupilumab (0.1, 1.0, or 10.0 μg/mL), in the presence of human IL-4 (50 pM) or IL-13 (50 nM) (Preprotech, USA). After 72 h, B cell activation was analyzed by flow cytometry, using CD23 or MHCII as activation markers (Biolegend, USA). Data were acquired with NxT software (Invitrogen, USA) and mean fluorescence intensity was analyzed.

### Ovalbumin-induced Th2 allergy mouse model

The ovalbumin-induced Th2 allergy mouse model is well characterized and validated^46,47^. B-hIL-4/hIL-4RA mice (Biocytogen, China) were sensitized with 200 mL intraperitoneal injections of 200 mg/mL ovalbumin (Sigma, USA) in PBS on Days 0, 7, and 14. Rademikibart or dupilumab (25 mg/kg) was subcutaneously administered on Days 20 and 23. On Days 21–25, mice inhaled atomized 2% ovalbumin for 30 min each day, triggering a Th2-driven recall response.

At termination, alveolar lavage fluid was collected, stained for CD45+ hematopoietic cells and eosinophils, and analyzed by flow cytometry. Markers comprised of mouse CD45 (Biolegend, USA), Siglec-F (BD Biosciences, USA), CD11c (eBioscience, USA), Ly6G (Biolegend), CD11b (BD Horizon, USA), CD16 (eBioscience, USA).

Serum samples were analyzed for ovalbumin-specific IgE via ELISA (Chondrex, USA).

Lung tissues (without lavage) were formalin fixed, paraffin embedded, sectioned (7 μm), and stained with hematoxylin and eosin. Histological changes (inflammatory cell infiltration around the blood vessels and bronchi, mucus in the bronchi and trachea, eosinophil infiltration around the blood vessels and bronchi) were scored by a pathologist unaware of the treatment groups. Scores were assigned in 0.5 increments ranging from 0 (no visual lesion) through to 1.5 (approximately 20% of the total area was affected).

### Exposure to rademikibart and dupilumab in wildtype mice

Exposure to rademikibart and dupilumab (5 mg/kg) in serum and BALF was determined at 4, 24, 48, 120, and 168 h post-treatment, based on medication-specific antibody concentrations in CD-1 wildtype mice (Beijing Vital River Laboratory Animal Technology, China). An in-house ELISA was used to evaluate the binding activity of rademikibart or dupilumab to sIL-4Rα using a Flex Station 3.

### Ex vivo human skin

Ex vivo human skin (ca. 750 μm thickness), from two independent adult donors without dermatological conditions, was sectioned using a 7 mm punch biopsy and placed into a Transwell insert, as previously as described^49^. Rademikibart or dupilumab were applied in cornification media (10 μg/mL). After overnight pretreatment, the media was vacuum-aspirated and fresh media was added containing stimulation cocktail (Th2; MedPharm, UK) and fresh therapeutic compound. The skin explants were harvested 24 h after commencement of stimulation, placed in RNAlater solution, and stored at 5 °C. RNA was isolated using the Kingfisher robotic RNA prep system (ThermoFisher, USA) and normalized prior to cDNA synthesis using the High Capacity cDNA Reverse Transcription Kit (Applied Biosystems, USA). Synthesized cDNA samples were diluted in RNase-free water, transferred to an optical 384-well plate (in duplicate), and TaqMan™ Gene Expression Master Mix (Applied Biosystems, USA) was added for RT-qPCR analysis of inflammation biomarkers.

qPCR values were calculated using the relative quantification approach. The Ct value (threshold cycle) is defined as the fractional cycle number at which the amount of amplified target reaches a fixed threshold. As qPCR run on a log2 scale, logarithmic calculations are used. Ct values were determined for target genes and a single normalizer gene (GAPDH) for each sample. To determine the Δ Ct for each sample, the Ct for the normalizer gene was subtracted from the Ct for the target gene. Data was normalized to the unstimulated skin sample.

### Study ethics and conduct

Research reported herein received ethics committee approval (Institutional Animal Care and Use Committee, Biocytogen, and MedPharm) prior to commencement and was performed by Connect Biopharma and MedPharm in accordance with relevant institutional and national guidelines and regulations.

Experiments with ex vivo human tissue were conducted in accordance with the Declaration of Helsinki; skin explants were obtained after the participants provided informed written consent, including for publication of the results. Reporting of animal experiments was in accordance with ARRIVE guidelines.

Animal studies were performed in Association for Assessment and Accreditation of Laboratory Animal Care (AAALAC)-accredited facilities. All animals were housed under specific pathogen-free conditions in individual ventilated cages and reared in line with standardized methods (20–26 °C, 40–70% humidity, 12‐hour light/dark cycle, free access to food and water).

### Statistical analysis

Calculations (K_D_, k_a_, k_d_, IC_50_, and EC_50_) and statistical analysis were performed using GraphPad Prism (GraphPad Software Inc, USA). Differences between groups were assessed by one-way ANOVA with Tukey’s multiple comparisons test. Statistical significance was defined by p < 0.05.

## Supplementary Information

Supplementary Information.

## Data Availability

The raw data supporting the conclusions of this article will be made available by the authors, without undue reservation (corresponding author: Xin Yang, DSc, xyang@connectpharm.com).
