# Supplementary material for: Preclinical immunological characterization of rademikibart (CBP-201), a next-generation human monoclonal antibody targeting IL-4Rα, for the treatment of Th2 inflammatory diseases
Source: Sci Rep. 2023 Jul 31;13:12411. doi: 10.1038/s41598-023-39311-2 (PMC10390583; doi:10.1038/s41598-023-39311-2)
Supplement: Supplementary file 1 — Supplementary Information. [file 41598_2023_39311_MOESM1_ESM.docx]

# Supplementary Figure 1. Rademikibart and dupilumab bind to distinct epitopes on human IL-4Rα


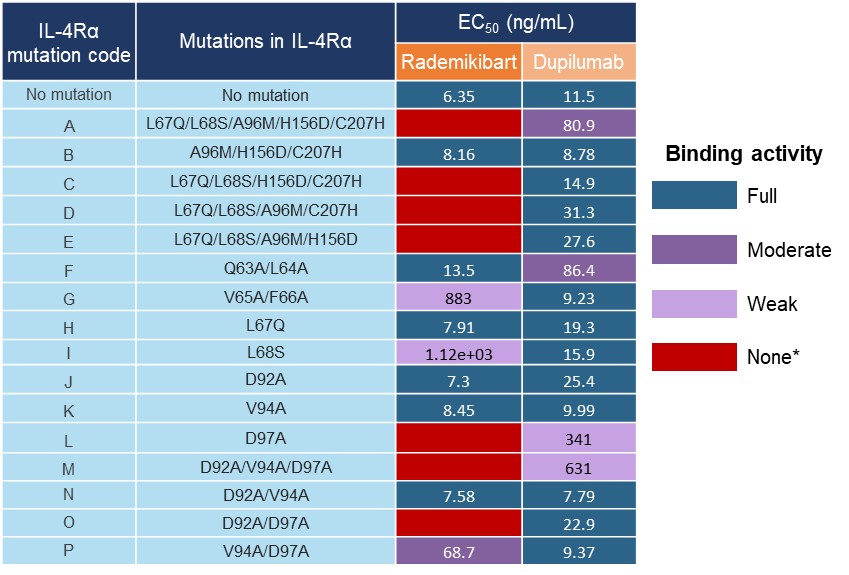
 EC_50_, half maximal effective concentration. *No binding activity detected up to the maximum antibody concentration of 2500 ng/mL.

# Supplementary Figure 2. Rademikibart inhibits, respectively, IL-4 and IL-13 induced (A and B) intracellular STAT6 signaling, (C and D) TF-1 cell proliferation, and (E and F) TARC secretion, generally with trends towards greater potency than dupilumab. IC_50_, half maximal inhibitory concentration.

#
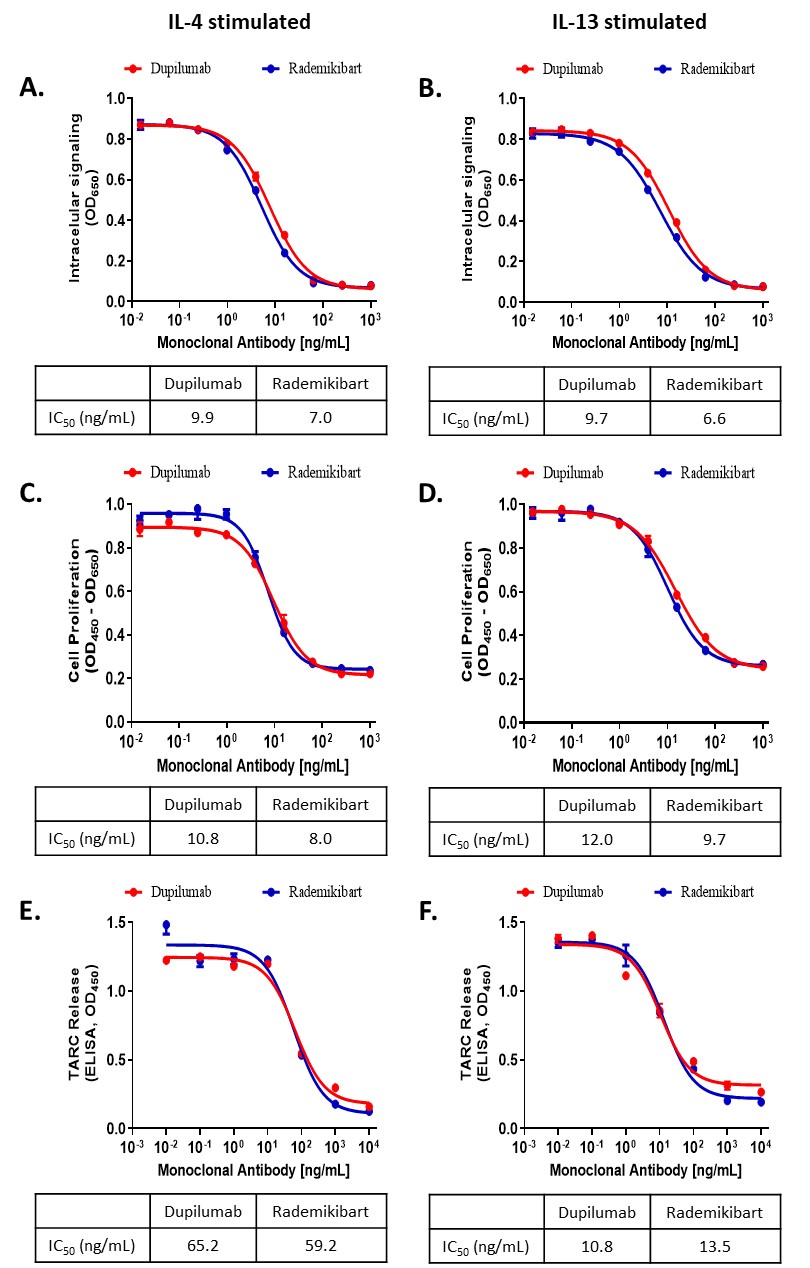


# Supplementary Figure 3. Rademikibart binds with high affinity to TF-1 cells. The findings were consistent with a non-significant trend towards higher potency with rademikibart versus dupilumab in the TF-1 cell proliferation assay (Table 2). FL1-H, fluorescence intensity.

#
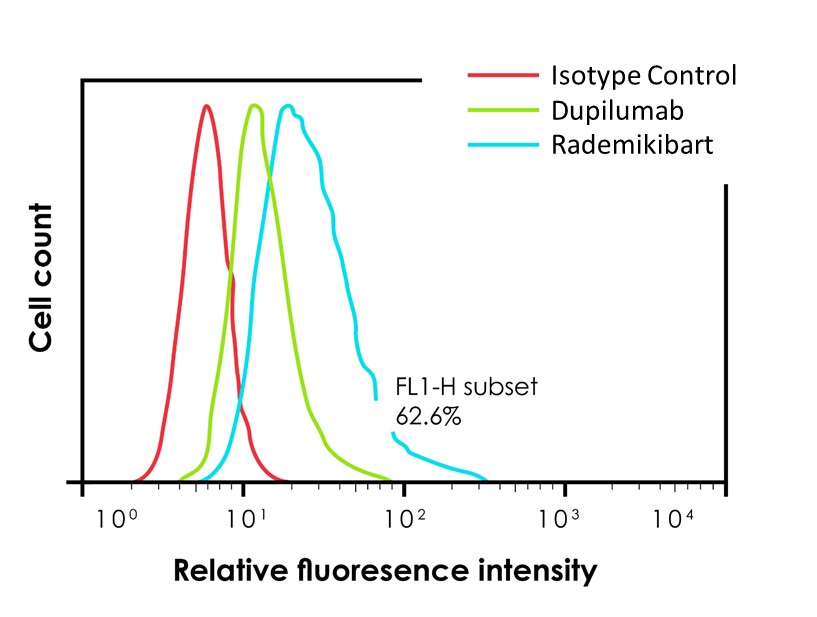


# Supplementary Methods. In-house ELISA protocol.

Phosphate Buffered Saline (PBS) solution (for use within 7 days of preparation) was used in PBST (500 µL Tween-20 in 1000 mL PBS). PBST was used in 1% and 2% BSA-PBST (bovine serum albumin [BSA] in PBST). PBST and BSA-PBST were prepared on the day of use.

The in-house ELISA procedure was as follows:

1. *Coating:* In-house manufactured sIL-4Rα-His (human, monkey, mouse, dog, rat, rabbit)* was diluted to 0.5 μg/mL in PBS, mixed by inversion, and added to a 96-well microplate (100 μL per well). The sealed microplate was incubated overnight at 2–8℃.
2. *Washing:* After discarding the solution, the microplate was patted dry on a clean paper towel, washed three times with PBS (300 μL per well), and patted dry again.
3. *Blocking:* 2% BSA-PBST was added to the 96-well microplate (300 μL per well). The sealed plate was incubated for 2 h ± 10 min at 37 ± 2℃.
4. *Washing:* The microplate was washed as in step 2.
5. *Addition of monoclonal antibody:* Rademikibart or dupilumab (a range of concentrations) were added to the 96-well microplate (100 µL per well). Each concentration of solution was added to multiple wells and 1% BSA-PBS was added as a blank control to one column. The sealed plate was incubated for 2 h ± 10 min at 37 ± 2℃.
6. *Washing:* The microplate was washed, initially three times with PBST (300 μL per well) and then, as in step 2, with PBS.
7. *Secondary antibody:* Anti IgG-Fc antibody (Sino Biological, China), diluted according to the supplier’s instructions in 1% BSA-PBS, was added using a multichannel pipette (100 μL per well).
8. *Washing:* The microplate was washed as in step 6.
9. *Color Development:* TMB substrate (100 μL per well) was added using a multichannel pipette (preincubated at 37 ± 2℃ for 10 minutes). The plate was incubated for 5-10 minutes at 37 ± 2℃.
10. *Stopping:* The reaction was terminated by adding 2M H_2_SO_4_ (50 µL per well) using a multichannel pipette.
11. *Reading:* Optical density absorption values were read at a wavelength of 450 nm using a Flex Station 3 microplate reader.

*Manufactured by inserting human, monkey (UniProt G7Q0S7), mouse (UniProt P16382), dog (UniProt A0A8P0NIW8), rat (UniProt Q63257-1) and rabbit IL-4Rα (UniProt A0A5F9DT58) ECD sequences into a pcDNA™3.1-His vector (ThermoFisher, USA), and then transfected Expi293F™ cells (ThermoFisher, USA). Recombinant proteins were purified using a Ni-NTA column.
